# Oncolytic alphavirus replicons mediated recruitment and activation of T cells

**DOI:** 10.1016/j.isci.2024.109253

**Published:** 2024-02-16

**Authors:** Darshak K. Bhatt, Saskia L. Meuleman, Baukje Nynke Hoogeboom, Toos Daemen

**Affiliations:** 1 Department of Medical Microbiology and Infection Prevention, University Medical Center Groningen, University of Groningen, 9713 AV Groningen, the Netherlands

**Keywords:** Immunology, Virology

## Abstract

Oncolytic viruses show promise in enhancing tumor immunogenicity by releasing immunogenic signals during tumor cell infection and lysis. In this study, we improved the virus-induced tumor immunogenicity of recombinant Semliki Forest virus (rSFV)-based replicon particles by encoding immunogenic cytokines such as C-X-C motif chemokine ligand 10 (CXCL10), FMS-like tyrosine kinase 3 ligand (Flt3L), or interferon-gamma (IFN-ƴ). Real-time imaging and flow cytometry of human cancer cell-based monolayer and spheroid cultures, using LNCaP or PANC-1 cells, revealed effective infection and transgene expression in both models. LNCaP cells exhibited higher and earlier rSFV infection compared to PANC-1 cells. While infected LNCaP cells effectively triggered immune recruitment and T cell activation even without encoding cytokines, PANC-1 cells demonstrated improved immune responses only when infected with replicons encoding cytokines, particularly IFN-ƴ, which enhanced tumor immunogenicity irrespective of cancer cell susceptibility to infection. Our study demonstrates that despite innate phenotypic disparities in cancer cells, rSFV-based replicons encoding cytokines can potentially generate effective immune responses in the tumor.

## Highlights

• rSFV-replicons effectively express transgenes in tumor monolayer and spheroids
• rSFV-replicons can induce transgene-independent immune recruitment and activation
• Innate disparities in antiviral response influence immune activation by tumor cells
• rSFV-replicons encoding cytokines enhance immune responses across tumor types

## Graphical abstract

**Figure:**
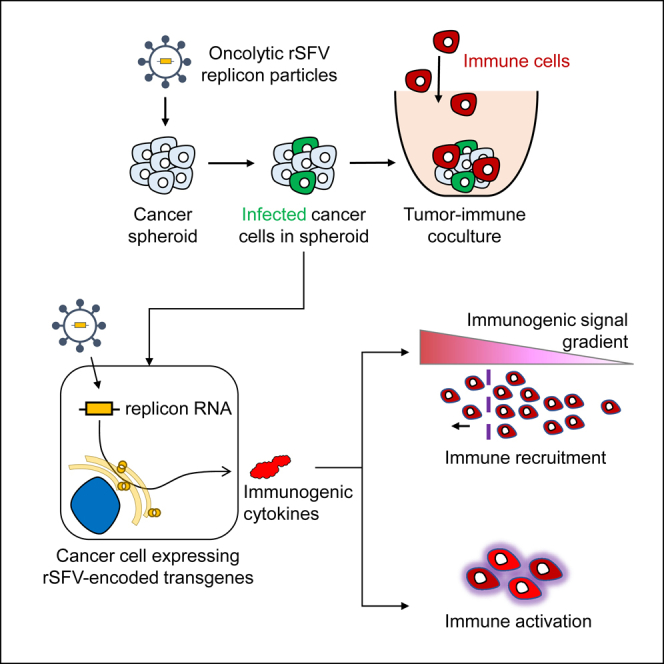

## Introduction

Cancer immunotherapy relies on modulating the host immune system to induce an anti-tumor response. Oncolytic viruses provide an innovative approach in this regard, not only due to their ability to infect and kill cancer cells but also because they aid in stimulating the host immune system and inducing an anti-tumor response.^1^^,^^2^,^3^^,^^4^ Engineering oncolytic viruses that are safe and potent in inducing strong anti-tumoral immune responses is therefore a promising strategy to improve therapeutic outcomes of cancer immunotherapy.^4^^,^^5^

**Figure 1:**
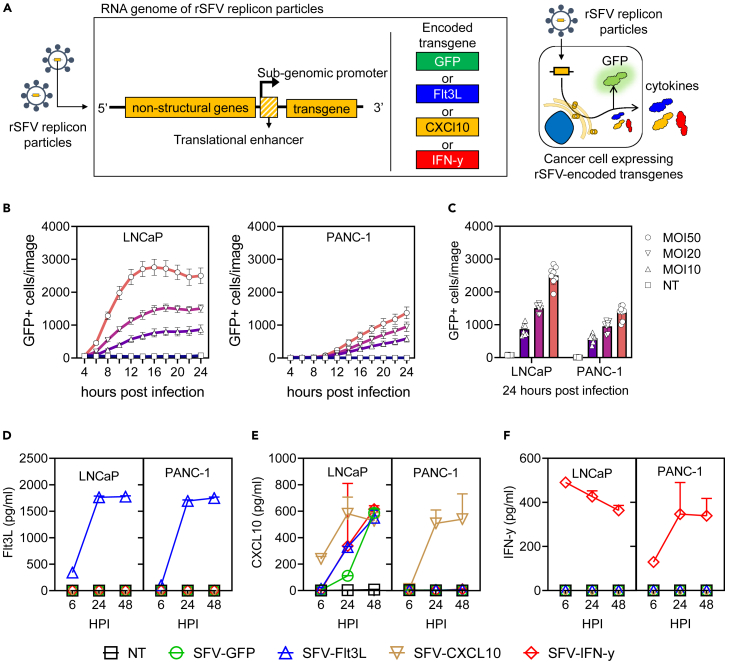
Kinetics of cytokine expression by rSFV-particles infected cancer cells in monolayer (A) Genetic design of rSFV-particles (left) capable of a single round of infection and enhanced cytokine expression (right). (B) Temporal kinetics of GFP expression by LNCaP and PANC-1 cell lines infected with SFV-GFP replicon particles at different multiplicity of infection (MOI). (C) Re-visualization of the data of GFP+ cells at 24 h post-infection. Production of extracellular Flt3L (D), CXCl10 (E), and IFN-ƴ (F) by LNCaP and PANC-1 cell lines, measured by ELISA after 6, 24, and 48 h of infection with SFV-replicon particles encoding different cytokines. See Figure S1 for microscopy images corresponding to the data depicted in (B and C). In (B and C) each plot represents data from 8 replicate-images. In (D, E, and F) the plots represent data from 2 experiments. Legend: NT, non-infected cancer cells; SFV-GFP, cancer cells infected with rSFV encoding GFP; SFV-Flt3L, cancer cells infected with rSFV encoding Flt3L; SFV-CXCL10, cancer cells infected with rSFV encoding CXCL10; SFV-IFN-ƴ, cancer cells infected with rSFV encoding IFN-ƴ. Data are presented as mean ± SEM.

To develop safe and potentially immunogenic oncolytic virotherapy, we engineered recombinant replicon particles based on Semliki Forest virus (SFV). SFV is a positive-strand RNA virus of the *Alphavirus* genus. The SFV genome is a replicon, meaning self-replicating as the viral RNA encodes so-called non-structural proteins responsible for RNA replication and translation. The design of recombinant SFV (rSFV) replicon particles involves the deletion of genes encoding the structural proteins of the virus and replacing them with transgenes of interest (Figure 1A). This simultaneously allows the production of high levels of transgene-encoded proteins but also generates safe, “suicidal” replicon particles capable of a single round of infection as they lack the genes encoding structural proteins.

Our group previously demonstrated that cancer vaccines based on rSFV replicon particles engineered to express tumor-associated antigens like E6 and E7 of human papillomavirus (HPV) and non-structural proteins of hepatitis-C virus (HCV) induce potent anti-tumoral immune responses.^6^^,^^7^ We recently also performed a phase 1 clinical trial with HPV-specific rSFV replicon vaccine demonstrating the feasibility, safety, and immunogenicity of this replicon vector system.^8^^,^^9^ A phase 2 trial in patients with a high-grade premalignant lesion of cervical cancer is now ongoing. The replicon particles of this cancer vaccine are injected intramuscularly. Others also studied the efficacy of intratumoral injections of SFV replicon particles as oncolytic virotherapy in murine tumor models. These replicons were engineered to express cytokines like XCL1, IL-12 or FMS-like tyrosine kinase 3 ligand (Flt3L), or checkpoint inhibitors and demonstrated strong T cell-dependent anti-tumor activity *in vivo*.^10^^,^^11^^,^^12^^,^^13^

Building upon these promising results, we aimed to investigate the potential of cytokine-encoding rSFV replicon particles to enhance immune responses in the context of human cancers. To assess the impact of these cytokine-encoding rSFV replicon particles, we conducted a comprehensive evaluation of their immunogenic profile. Our primary measure of interest was the extent to which virus-infected cells could recruit and activate immune cells in two distinct experimental setups. First, we utilized monolayer co-cultures of human cancer cells with immune cells, which allowed us to study the immune response in a controlled and simplified environment. Subsequently, to better mimic the tumor microenvironment and its complexities, we utilized spheroids of human cancer cells in our evaluations.

## Results

As effective immune responses to oncolytic virotherapy depend on various factors, we investigated rSFV replicon particle-induced immune responses (1) dependent on encoded cytokines, (2) in different target cancer cells, and (3) in the context of cancer cell monolayer or spheroid-based models. We encoded individual cytokines in the SFV replicon genome with the aim to induce recruitment and activation of immune cells: C-X-C motif chemokine ligand 10 (CXCL10) was selected as a recruitment signal and Flt3L and interferon-gamma (IFN-ƴ) as recruitment and activation signals. Notably, these cytokines have been described previously to be associated not only with an immunogenic tumor-microenvironment but also with better prognosis.^14^^,^^15^^,^^16^^,^^17^^,^^18^^,^^19^^,^^20^ Cancer cell lines (LNCaP and PANC-1) were selected based on their characteristic ability to form spheroids independent of scaffold-matrix, and their HLA-2A positive phenotype to allow co-culture assays with peripheral blood mononuclear cells (PBMC) from HLA-2A typed healthy donors.

### Transgene expression by cancer cell monolayers infected with recombinant Semliki Forest virus particles

Through time-lapse microscopy, we first assessed if LNCaP and PANC-1 cancer cells could be infected by rSFV replicon particles encoding the reporter gene for green fluorescent protein (rSFV-GFP). We observed that upon infection with rSFV-GFP particles, both LNCaP and PANC-1 cells stably expressed GFP. GFP expression in LNCaP cells occurred earlier and in a higher percentage of cells (Figure 1B) as compared to PANC-1 cells (Figures 1C and S1). Upon infection with rSFV particles encoding CXCL10, Flt3L or IFN-ƴ, both LNCaP and PANC-1 cells were observed to produce and secrete high amounts of the respective cytokines, detectable from 6 h post-infection (Figures 1D–1F). As expected, non-infected LNCaP or PANC-1 cells did not produce detectable amounts of either CXCL10, Flt3L or IFN-ƴ. Notably, CXCL10 expression was observed in LNCaP cells infected with rSFV particles encoding either of the transgenes studied (Figure 1E).

### Immune recruitment by cancer cell monolayers infected with rSFV particles

**Figure 2:**
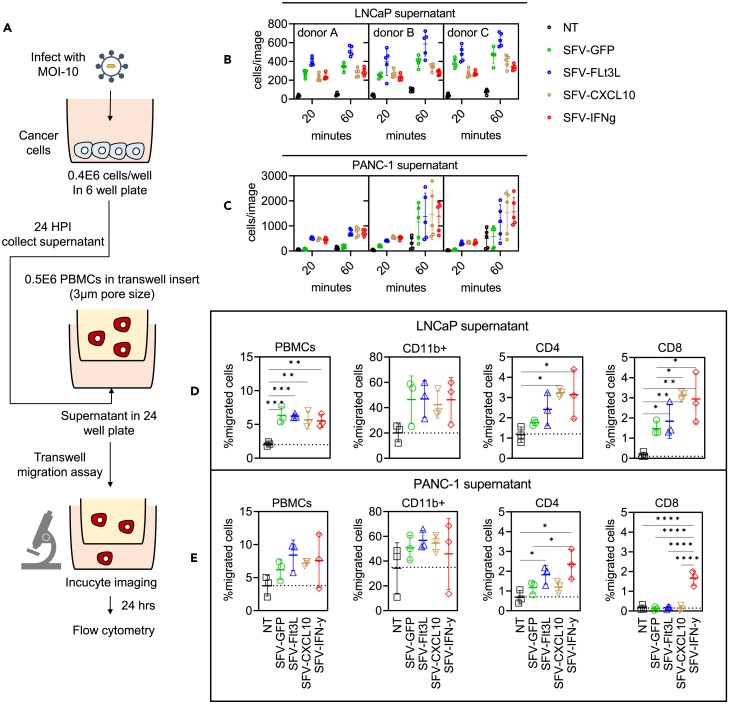
Kinetics of PBMC migration toward secreted signals from rSFV-infected cancer cells (A) The setup of a Transwell assay to assess the recruitment of immune cells toward the supernatants of SFV-infected cancer cells using microscopy. Temporal kinetics of PBMC migration (B and C) and flow cytometry-based endpoint comparison (D and E) between supernatants derived from infected or non-infected LNCaP cells or PANC-1 cells. See Figure S2 for the microscopy images corresponding to the data depicted in (B–E). PBMC from healthy donors were used for the experiment. Each condition had 15 replicates derived from 5 images of 3 independent donors. Data are presented as mean ± SEM. A p value of 0.05 was considered a statistically significant difference between compared groups (∗ = p < 0.05, ∗∗ = p < 0.01 and, ∗∗∗ = p < 0.001).

To assess if rSFV-infected cells induced recruitment of immune cells we determined the level of migration of PBMC through membrane pores of a transwell filter system toward 24-hour-culture supernatants harvested from uninfected or rSFV-infected LNCaP and PANC-1 cells (Figure 2A). Low levels of migration of PBMCs toward supernatants from non-infected cells were observed for PANC-1 but not for LNCaP cells. Migration of PBMC toward the supernatant of both SFV-infected LNCaP and PANC-1 cells occurred in less than 20 min (Figures 2B and 2C). PBMC migration was observed for all SFV-infected LNCaP (Figure 2B) and PANC-1 cell supernatants (Figure 2C), including infection with control particles SFV-GFP. Yet, in two of the three PBMC populations migration of PBMC toward supernatants from infected PANC-1 cells expressing cytokines was higher compared to SFV-GFP-infected cell supernatants. After 24 h, we evaluated the migrated PBMC populations using flow cytometry. In both LNCaP (Figure 2D) and PANC-1 cells (Figure 2E), encoding cytokines led to an additional increase in migration of overall PBMCs, which was significantly high for CD4 and CD8 T cells, but not CD11b myeloid cells.

### Activation of T cells by cancer cell monolayers infected with rSFV particles

**Figure 3:**
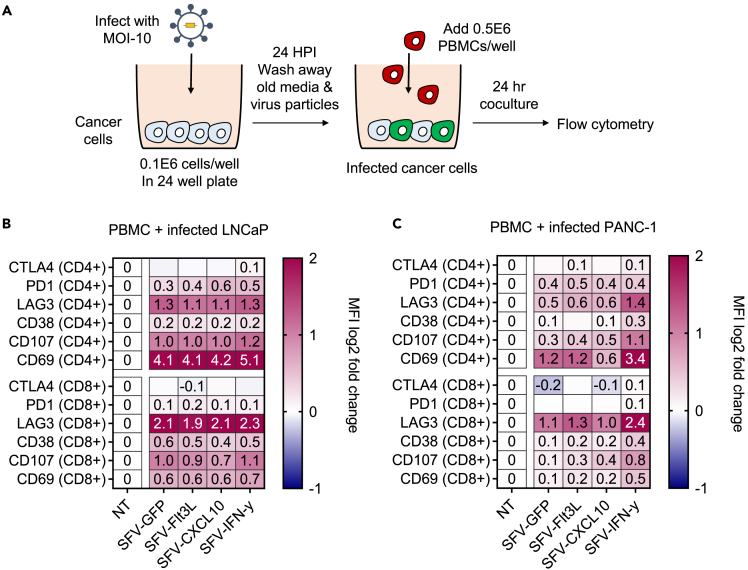
Immune activation by rSFV-infected cancer cells (A) The setup of a monolayer-based co-culture assay of infected cancer cells and PBMC to assess the immunogenic potential of rSFV-particles. Protein level expression of exhaustion and activation markers in CD4^+^ or CD8^+^ T cells upon co-culture with infected or non-infected (B) LNCaP or (C) PANC-1 cells. The median fluorescence intensity (MFI) for each marker quantified through flow cytometry was normalized to the non-infected (NT) condition. The values in (B) and (C) show the mean expression values of different donors represented on a log2 scale. See Figure S3 for the gating strategy and visualization of expression levels of individual markers and data of different donors for respective conditions. In (B and C) the plots represent data from duplicate conditions of 3 independent healthy donors.

We measured T cell activation through a co-culture of PBMCs and rSFV-infected cancer cells (Figure 3A). Specifically, in co-cultures with rSFV-infected LNCaP and PANC-1 cells, CD4 and CD8 T cells were found to exhibit a pro-inflammatory profile represented by an increased expression of the activation markers CD69 and CD107 compared to controls with non-infected cancer cells (NT) (Figures 3B and 3C). The expression of the exhaustion marker LAG3 (lymphocyte-activation gene 3) was upregulated on T cells in response to a co-culture with infected cells, while PD1 (programmed cell death protein 1) and CTLA4 (cytotoxic T-lymphocyte associated protein 4) expression did not change. Notably, the co-cultures with infected LNCaP cells induced T cell activation independent of the expressed transgene (Figure 3B). For PANC-1 cells, significant T cell activation was only observed upon co-culture with replicon-infected cells expressing IFN-ƴ (Figure 3C). Remarkably, the exhaustion marker LAG3 showed a higher upregulation in CD8 T cells compared to CD4 T cells. This may be attributed to the robust activation of CD8 T cells in the non-infected condition, as evidenced by the elevated expression of CD69 on T cells in the absence of infection (Figure S3).

### Transgene expression in cancer spheroids infected with rSFV particles

**Figure 4:**
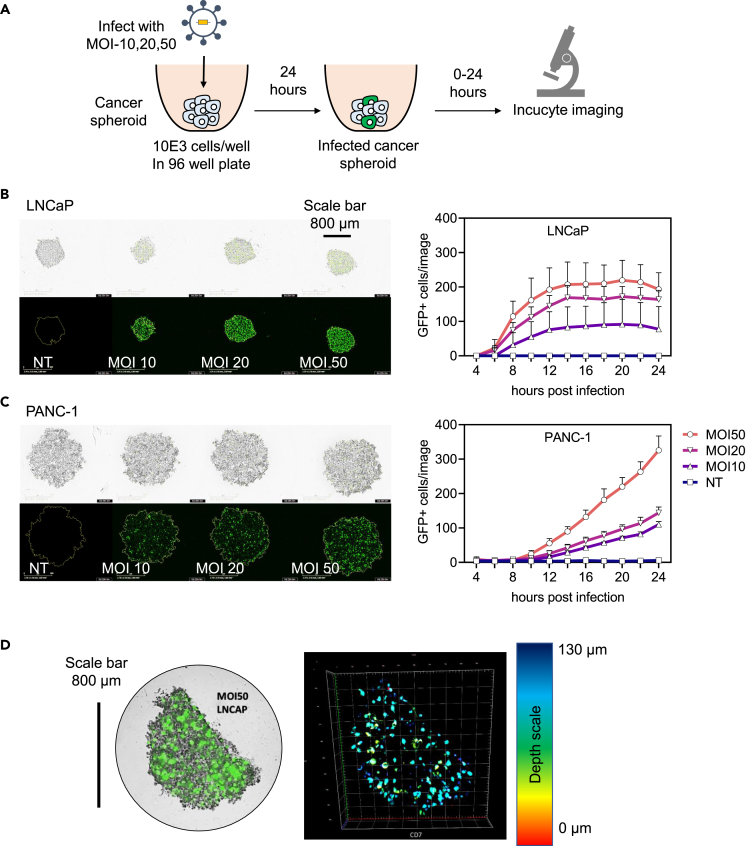
Kinetics of transgene expression by rSFV-infected cells in cancer-spheroids (A) The setup of the cancer-spheroid generation and infection assay to measure transgene (GFP) expression using microscopy. Representative images of cancer-spheroids generated from LNCaP (B) and PANC-1 cells (C), and microscopy-based temporal quantification of GFP+ cells in cancer-spheroids from LNCaP and PANC-1 cells (on the right). (D) Confocal microscopy-based characterization of spatial information of GFP+ cells present in an LNCaP spheroid (on the left). The depth at which a GFP-expressing cell is present in the spheroid is depicted by the color-coded legend (on the right). In (B and C) the top row depicts brightfield images, the bottom row depicts fluorescence images for GFP visualization, and the plots represent data from 8 replicate-spheroids. See Figure S4 for microscopy images corresponding to (B and C). Data are presented as mean ± SEM.

We established a spheroid-based assay to evaluate the potential of rSFV particles to enhance immune responses in a 3D model. Using time-lapse microscopy, we first assessed if cancer spheroids derived from LNCaP or PANC-1 cells could be infected and if replicon-encoded transgenes were expressed in detectable quantities (Figure 4A). LNCaP and PANC-1 spheroids were observed to have innate differences in morphology and cell organization; LNCaP-spheroids were more compact and smaller as compared to the loose and large PANC-1-spheroids (Figures 4B and 4C, brightfield images). Despite morphological differences, both spheroids could be infected as indicated by the expression of GFP (Figures 4B and 4C, fluorescence images). Similar to the monolayer-experiment, the number of GFP-positive cells could be observed earlier in LNCaP-spheroids compared to PANC-1 spheroids (Figures 4B and 4C). Through confocal microscopy, GFP-positive cells were also found to be present within the mass of the spheroid and not only at the periphery (Figure 4D).

### Immune recruitment by cancer-spheroids infected with rSFV particles

**Figure 5:**
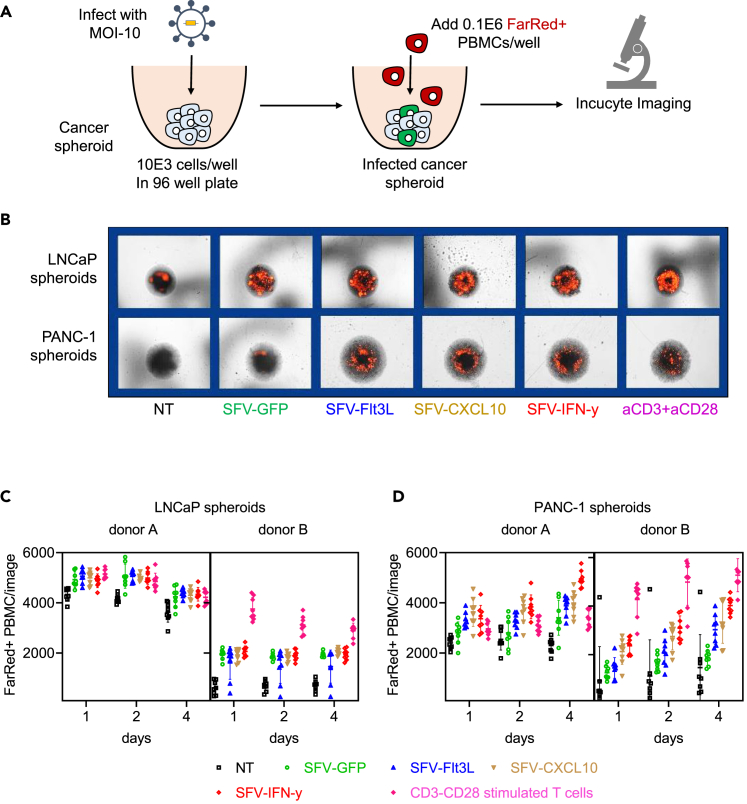
Infiltration-kinetics of PBMC toward rSFV-infected cancer-spheroids (A) The setup of a cancer-spheroid and PBMC co-culture experiment to quantify immune infiltration in a 3D model using microscopy. (B) Representative microscopy images of far-red stained PBMC (in red) associated with LNCaP (top row) or PANC-1 (bottom row) spheroids at day 4 post co-culture. Kinetics of PBMC infiltration in (C) LNCaP or (D) PANC-1 spheroids infected with rSFV-particles up to 4 days post co-culture. See Figure S5 to visualize the microscopy images of different replicates for respective conditions. As a positive control, T cells stimulated overnight with antibodies against CD3 and CD28 were used. In (B and C) the plots represent data from 8 replicate-spheroids and 2 independent donors. The number of far-red positive PBMC is quantified as the number of red events per image, where each image consists of an individual spheroid per well. Data are presented as mean ± SEM.

We used the spheroid-based 3D model to study the recruitment of PBMC-derived immune cells toward and into the spheroids (Figure 5A). Microscopy-based counting indicated an increased number of FarRed stained PBMC associated with both LNCaP- and PANC-1-spheroids over time (Figure 5B). With LNCaP-spheroids, replicon-infected spheroids had a higher PBMC count as compared to non-infected spheroids, which was irrespective of the transgene expressed. (Figure 5C). Whereas, with PANC-1-spheroids, replicon-infected spheroids had a higher PBMC count while furthermore, expression of IFN-ƴ enhanced this effect (Figure 5D). Notably, this increase in PBMC count associated with the spheroids was comparable to that of T cells stimulated overnight with CD3-CD8 antibodies prior to co-culture.

### Activation of T cells by cancer spheroids infected with rSFV particles

**Figure 6:**
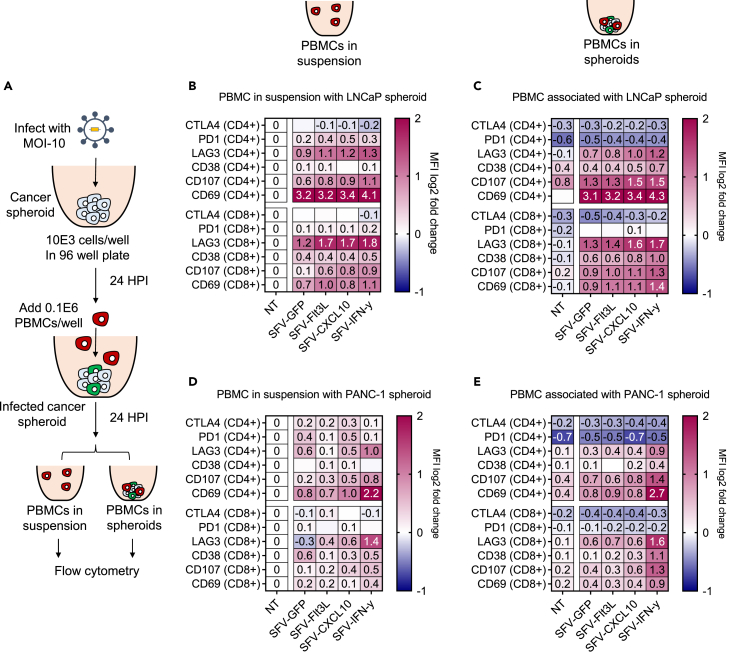
Immune activation by rSFV-infected cancer-spheroids (A) The setup of cancer-spheroid and PBMC co-culture to assess the immunogenic potential of rSFV-particles in a 3D model. Protein level expression of exhaustion and activation markers assessed by flow cytometry in CD4^+^ or CD8^+^ T cells upon co-culture with infected or non-infected (B and C) LNCaP or (D and E) PANC-1-spheroids. (B) and (D) represent the expression profile of CD4^+^ or CD8^+^ T cells present outside the spheroid in suspension media, while (C) and (E) represent the expression profile of T cells associated with or present in the spheroids. The median fluorescence intensity (MFI) for each marker quantified through flow cytometry is normalized to the non-infected (NT) condition from the T cells present in suspension. The values in (B–E) are the mean expression values of different donors represented on a log2 scale. In (B–E) the plots represent data from duplicate conditions of 3 independent healthy donors. See Figure S6 for the visualization of expression levels of individual markers and data of different donors for respective conditions. The gating strategy is the same as in Figure S3A.

Using the spheroid-based co-culture model, we investigated the potential of rSFV-infected cancer cells to induce T cell activation within the spheroid. Specifically, we assessed the difference in phenotypes of CD4 and CD8 T cells that were found associated with the spheroids in reference to those found in suspension outside the spheroids (Figure 6A). Co-culture with replicon-infected LNCaP-spheroids (Figure 6B) or specifically SFV-IFN-ƴ-infected PANC-1-spheroids (Figure 6C) upregulated activation markers CD69 and CD107, on CD4 and CD8 T cells present in suspension or associated with spheroids. There was little difference in the transgene-dependent activation/exhaustion phenotypic profile of T cells associated with different replicon-infected LNCaP-spheroids. Whereas, in SFV-IFN-ƴ infected PANC-1-spheroids a significant change in activation/exhaustion phenotype was observed, indicating IFN-ƴ dependent immunogenic effects.

## Discussion

Immune recruitment and activation are often considered as a critical hallmark of immunogenic tumors and a prognostic factor of effective cancer immunotherapy.^21^^,^^22^^,^^23^ We therefore set out to engineer a safe and effective SFV-based oncolytic virotherapy aimed to enhance the immunogenicity of the tumor microenvironment. Utilizing human-based monolayer and 3D cancer models, we showed that rSFV replicon particles can efficiently express high levels of encoded cytokines in target cancer cells, leading to robust immune recruitment and activation in both models. Importantly, our findings indicate that infection with rSFV replicon particles can induce potent immune responses, which can be further improved by encoding multifunctional cytokines such as IFN-γ. These results highlight the potential of our engineered virotherapy as a promising strategy to promote immunogenicity in tumors, potentially contributing to more effective cancer immunotherapy outcomes.

Even without encoding immunogenic cytokines (SFV-GFP replicon particles), rSFV-infected cancer cells induced strong migration of PBMC through a transwell and caused upregulation of CD4 and CD8 T cell degranulation (CD107) and differentiation (CD69) markers in cancer cell monolayers. A similar effect was observed in the attraction of PBMC and activation of CD4 and CD8 T cells in the spheroid-based 3D model. Interestingly, both LNCaP and PANC-1 rSFV-infected cells induced migration of myeloid cells (CD11b+ cells) independent of cytokine expression. These results are supported by various studies that describe SFV-replicon particles to be natively capable of initiating immune responses by causing cancer cell death coupled with release of RNA transcripts activating Toll-like receptors and type-I interferon responses.^24^^,^^25^ Moreover, SFV-induced oncolysis is known to increase availability of cancer antigens and expression of co-stimulatory molecules leading to an efficient antigen-presentation for an adaptive immune response.^12^^,^^13^

Although rSFV-infected LNCaP and PANC-1 cells generated immune responses independent of encoded transgenes, our research also revealed that encoding cytokines could enhance these responses. For PANC-1 cells, especially infection with SFV-IFN-ƴ induced a significantly stronger immune response in the recruitment and activation of T cells compared to other rSFV replicon particles. The different responses between LNCaP and PANC-1 cells may, apart from the origin and other intrinsic differences, be due to the lack of an active interferon signaling pathway in LNCaP cells.^26^ Conversely, PANC-1 cells can actively initiate interferon-mediated antiviral responses. To unravel if the differences between the cell lines could be ascribed to this antiviral response, we also blocked the interferon pathway in PANC-1 cells by ruxolitinib (Figure S7A), which only modestly enhanced the number of GFP expressing PANC-1 cells upon infection with SFV-GFP (Figure S7B). Moreover, the increase in the number of GFP-expressing cells did not result in enhanced immune activation by rSFV-infection alone and still depended on the expression of IFN-y by infected PANC-1 cells (Figures S7C, S7D, and S8). This suggests the possibility of other phenotypic differences between these cell lines such as variations in viral entry and replication, antigen presentation, immunomodulatory factors, microenvironment influences, and genetic variations which may contribute to the observed differences in immune activation upon rSFV infection.

Innate disparities in the response to viral infection are evident between LNCaP and PANC-1 cells, as demonstrated by distinct baseline gene expressions of antiviral immune proteins (Figure S10A). Notably, LNCaP cells exhibit downregulated JAK1 and STAT1 compared to PANC-1 cells, potentially influencing their differential responses upon infection. Additionally, upon SFV-GFP infection, LNCaP cells exhibit significantly higher expression of IFN-β and CXCL10 than PANC-1 cells (Figure S10B), which could be explained by the elevated levels of RIG-I or TLR3 expression in LNCaP, leading to better RNA-sensing and boosting of subsequent immune responses. These observations also suggest that in addition to transgenic-cytokines, there may also be differences in innate host-encoded cytokines and immunomodulatory factors between LNCaP and PANC-1 cells which can also influence immune recruitment and activation. These innate differences between LNCaP and PANC-1 also influence the expression of rSFV-encoded transgenes, with Flt3L, CXCL10, and IFN-γ concentrations being notably higher in LNCaP than PANC-1 at 6 h post-infection. This agrees with the kinetics of GFP expression in these cell lines, where LNCaP cells start expressing GFP earlier than PANC-1 cells. Despite initial disparities, the levels and kinetics of respective transgenic expression between the two cell lines converge at 24 and 48 h. Finally, variations in the kinetics of respective cytokines may stem from innate differences in protein folding, secretion efficacy, and individual cytokine half-lives. Surprisingly, LNCaP cells express CXCL10 upon SFV-replicon particle infection independent of encoded transgenes, with detectable expression at 6 h, suggesting innate cellular induction. This unexpected finding challenges conventional understanding, as CXCL10 is typically associated with interferon signaling, which is compromised in LNCaP cells lacking JAK1 gene expression (Figure S10A and Dunn et al.^26^). Our qPCR analysis reveals significantly elevated RIG-I and TLR3 expression in LNCaP cells compared to PANC-1 upon rSFV-infection even in the absence of transgenic-CXCL10 expression (Figure S10B). This aligns with higher cellular CXCL10 mRNA expression in LNCaP cells compared to PANC-1 upon rSFV infection, supporting the hypothesis that innate non-conventional cellular pathways, such as NF-kB or IRF3 activation,^27^^,^^28^^,^^29^ play a role in SFV-induced CXCL10 expression independent of the JAK-STAT pathway. Nevertheless, encoding cytokines in rSFV replicon particles can induce a robust release of cytokines and enhance the immune response, irrespective of cancer cell sensitivity to oncolysis. In this way, inducing a stronger immune response by encoding relevant immunogenic transgenes can be a complementary mechanism to increase tumor immunogenicity. This is relevant considering that more than 80% of cancer samples from patients were found not to harbor defects in antiviral signaling (Figure S9)^30^ or may even exhibit other unexplored resistance mechanisms.^31^

Encoding specific cytokines in rSFV-replicon particles enhances tumor immunogenicity; however, the outcomes of such immune modulation can vary based on the chosen cytokines. Our results show that encoding CXCL10 and Flt3L predominantly enhance immune recruitment rather than immune activation, as observed in the case of IFN-y. CXCL10, also known as IFN-ƴ-induced protein 10 (IP-10), acts as a chemoattractant for immune cells like T cells and natural killer (NK) cells.^32^^,^^33^ By encoding CXCL10, rSFV-replicon particles can enhance the recruitment of these immune cells to the tumor site, creating a more favorable environment for immune responses. Similarly, Flt3L is known to increase the production and maturation of dendritic cells, crucial players in initiating immune responses.^34^ However, while these cytokines facilitate immune cell migration and maturation, they might not directly activate these cells to the extent needed for robust antitumor immune responses.^12^^,^^35^^,^^36^ In contrast, encoding IFN-y in rSFV-replicon particles can trigger more direct and potent immune activation. IFN-y is a key cytokine that stimulates multiple immune pathways,^37^ including enhancing antigen presentation,^38^ boosting T cell cytotoxicity,^39^ and promoting inflammatory responses.^37^^,^^40^^,^^41^ These mechanisms collectively lead to a more comprehensive immune activation against cancer cells. Therefore, while CXCL10 and Flt3L play vital roles in recruiting immune cells to the tumor microenvironment, they might require additional cytokines or signals to induce full immune activation. In contrast, IFN-gamma’s ability to directly stimulate various immune mechanisms makes it a more potent activator of antitumor immune responses.

Our findings regarding the activation of immune responses by rSFV are consistent with previously reported observations. Especially, Smerdou and group have demonstrated that rSFV-particles expressing IL-12 or a combination of XCL1 and Flt3L can induce strong T cell-dependent anticancer activity in murine tumor models, which could be further enhanced by immune-checkpoint blockade.^12^^,^^13^ Importantly, these studies have shown that rSFV-particles can successfully induce local and systemic antitumor immunity in animal models, thus supporting the promise of a potential cancer immunotherapy in the clinic. Likewise, different oncolytic viruses engineered to carry immunogenic molecules, such as chemokines, cytokines, T cell-engagers, or checkpoint-blocking antibodies, have provided evidence supporting the approach of enhancing tumor immunogenicity to enhance therapeutic outcomes.^3^^,^^5^^,^^42^

The primary focus of our study was to study oncolytic virus induced immune recruitment and activation, which provides groundwork for further studies to also include assessment of antitumor activity. In future perspectives, it would be beneficial to explore additional factors such as the influence of the immune microenvironment, which may influence the antitumor response generated by oncolytic viruses. Expanding the assessment to include the impact of rSFV replicon particles on other immune cell types, such as NK cells, and dendritic cells, could provide valuable insights. Additionally, conducting a head-to-head comparison of immune responses generated between spheroid-based models and *in vivo* humanized murine models with xenograft tumors could offer a more systemic and comprehensive evaluation. Moreover, utilizing patient-derived organoids as an alternative model system may be crucial in elucidating the variability in rSFV particle-induced immune responses, considering the potential inter-patient and intra-tumoral heterogeneity. By addressing these aspects, future studies can contribute to refining the understanding and potential applications of oncolytic virotherapy for improving cancer immunotherapy outcomes.

In summary, our results describe that the expression of immunogenic cytokines results in an additive effect on the immunogenic potential of rSFV-particles. rSFV-mediated infection and oncolysis release inflammatory signals that cause rapid recruitment of immune cells and effective T cell activation in cancer monolayer and spheroid-based models. Furthermore, these insights open up exciting avenues toward more safe, effective, and universal approaches to combat cancer using oncolytic virotherapy.

### Limitations of the study

The immune-stimulatory effects of rSFV replicon particles encoding cytokines was demonstrated in tumor-immune spheroid and monolayer co-cultures. The antitumor killing mediated by the immune cells remains to be explored. Moreover, the study does not fully consider the intricate influence of the immune microenvironment, and our emphasis on T cell responses may not fully capture the broader spectrum of immune cell dynamics. Expanding the study to address these gaps and to assess rSFV efficacy across different tumor types in patient derived organoids holds potential.

## STAR★Methods

### Key resources table

**Table:** 

REAGENT or RESOURCE	SOURCE	IDENTIFIER
**Antibodies**		

PE anti-human CD223 (LAG-3) 11C3C65	Biolegend	369306
Brilliant Violet 421™ anti-human CD152 (CTLA-4) BNI3	Biolegend	369606
APC anti-human CD279 (PD-1) A17188B	Biolegend	621610
PE anti-human CD107a H4A3	Biolegend	328608
APC anti-human CD69 FN50	Biolegend	310910
Brilliant Violet 421™ anti-human CD38 HIT2	Biolegend	303526
APC/Cyanine7 anti-human CD8 SK11	Biolegend	344714
FITC anti-human CD4 OKT4	Biolegend	317408

**Bacterial and virus strains**		

Plasmid (Helper-2 and SFV-transgene) constructs to produce recombinant SFV replicon particles	Liljestrom lab, Karolinska Institutet Sweden	NA
*E coli* K12 JM110	Agilent	200239

**Biological samples**		

Human buffy coats for PBMC isolation from HLA2A-typed healthy donors	Sanquin, Nijmegen, The Netherlands	NA

**Chemicals, peptides, and recombinant proteins**		

Restriction enzyme PspOMI	ThermoFischer Scientific	ER0131
Restriction enzyme XmaI	ThermoFischer Scientific	ER0171
Restriction enzyme SpeI	ThermoFischer Scientific	ER1251
SP6 polymerase	Amersham Pharmacia Biotech, Piscataway, US	NA
α-chymotrypsin	Sigma Aldrich	C4129
Aprotinin	Sigma Aldrich	A1153
Celltrace FarRed dye	Invitrogen	C34572
Ruxolitinib	Stem Cell Technologies	73402
TRIzol RNA extraction reagent	Thermofischer Scientific	15596018

**Critical commercial assays**		

ELISA kit for CXCL10	Biolegend	439904
ELISA kit for Flt3L	RnD systems	DFK00
ELISA kit for CXCL10	Biolegend	430101
TB Green® Premix Ex Taq™ (Tli RNase H Plus)	TAKARA	RR420W
PrimeScript™ RT Reagent Kit with gDNA Eraser (Perfect Real Time)	TAKARA	RR047A

**Experimental models: Cell lines**		

Hamster, BHK-21 cells	ATCC	ccl-10
Human, LNCaP cells	ATCC	crl-1740
Human, PANC-1 cells	ATCC	crl-1469

**Oligonucleotides**		

Human nfkb1 gene Forward: GCAGCACTACTTCTTGACCACC Reverse: TCTGCTCCTGAGCATTGACGTC	This paper	NA
Human stat1 gene Forward: ATGGCAGTCTGGCGGCTGAATT Reverse: CCAAAACCAGGCTGGCACAATTG	This paper	NA
Human ddx58 (RIG-I) gene Forward: CACCTCAGTTGCTGATGAAGGC Reverse: GTCAGAAGGAAGCACTTGCTACC	This paper	NA
Human tlr3 gene Forward: GCGCTAAAAAGTGAAGAACTGGAT Reverse: GCTGGACATTGTTCAGAAAGAGG	This paper	NA
Human myd88 gene Forward: GAGGCTGAGAAGCCTTTACAGG Reverse: GCAGATGAAGGCATCGAAACGC	This paper	NA
Human cxcl10 gene Forward: GGTGAGAAGAGATGTCTGAATCC Reverse: GTCCATCCTTGGAAGCACTGCA	This paper	NA
Human ifn-beta1 gene Forward: GGTTACCTCCGAAACTGAAGA Reverse: CCTTTCATATGCAGTACATTAGCC	This paper	NA
Human gapdh gene Forward: GTCTCCTCTGACTTCAACAGCG Reverse: ACCACCCTGTTGCTGTAGCCAA	This paper	NA

**Recombinant DNA**		

Human CXCL10 gene	This paper	NA
Human IFN-gamma gene	This paper	NA
Human Flt3L gene	This paper	NA

**Software and algorithms**		

Graphpad Prism	Graphpad	https://www.graphpad.com/
Incucyte® Software Modules	Sartorious	https://www.sartorius.com/
FlowJo	FlowJo	https://www.flowjo.com/

**Other**		

Transwells 3 μm pore size	Grenier	662630
Nunclon Sphera 96 well plates	ThermoFischer Scientific	174925

### Resource availability

#### Lead contact

Further information and requests for resources and reagents should be directed to and will be fulfilled by the lead contact Toos Daemen (c.a.h.h.daemen@umcg.nl).

#### Materials availability

All unique reagents generated in the study are available upon request.

#### Data and code availability

• All data reported in this paper will be shared by the lead contact upon request.
• This paper does not report original code.
• Any additional information required to reanalyze the data reported in this paper is available from the lead contact upon request.

### Method details

#### Cell culture

Cancer cells (LNCaP, PANC-1) or BHK21 cells were cultured at 37°C with 5% CO_2_ in RPMI medium (Life Technologies, Paisley, UK) supplemented with 10% fetal bovine serum and 100 U/ml penicillin (Life Technologies) and 100 μg/ml streptomycin (Life Technologies). This media composition is referred to later as complemented media. The culture and passage of the LNCaP cell line was always done in flasks or plates pretreated with 0.01% of poly-l-lysine. PBMC were similarly cultured in RPMI medium, supplemented with 10% fetal bovine serum and 100 U/ml streptomycin and penicillin.

#### Design of rSFV-particles

The helper RNA system developed previously by Smerdou and Liljestrom allows the production of rSFV-replicon particles that are capable of expressing transgenes and of a single round of infection.^43^ Our lab has previously adopted this rSFV system to engineer high-level antigen expression for safe therapeutic vaccines.^44^ Using this system as a starting point, we designed three different rSFV-replicons that encode for a single cytokine - Flt3L, IFN-ƴ, and CXCL10 respectively. Similarly, we also designed rSFV-particles encoding enhanced green fluorescent protein (EGFP). Briefly, the transgenes were ordered as a DNA construct (Eurofins Genomics, Ebensburg, Germany) and were cloned in the SFV-replicon backbone plasmid (pSFV) by using PspOMI and XmaI as restriction sites and *E. coli* JM110 as the competent cell chassis. Furthermore, Sanger sequencing was performed on isolated colonies to validate cloning (Eurofins Genomics). A schematic diagram of SFV-replicon expressing cytokines is shown in Figure 1A.

#### rSFV-particle production

pSFV containing respective transgenes and a SFV-Helper2 plasmid were linearized by digestion with SpeI (Life Technologies) for RNA synthesis by SP6 polymerase (Amersham Pharmacia Biotech, Piscataway, US) mediated *in vitro* transcription in the presence of capping analog (Life Technologies). Subsequently, pVREP RNA and SFV-Helper-2 RNA were mixed in a 2:1 ratio and co-transfected in BHK-21 cells in the presence of electroporation buffer using the BioRad Gene Pulser II (2 pulses of 850 V / 25 μF; BioRad, Hercules, US). After electroporation, the cells were cultured in RPMI-1640 medium supplemented with 5% (v/v) fetal calf serum (FCS), 100 U/ml penicillin and 100 μg/ml streptomycin for 48 hours at 30°C with 5% CO_2_. The rSFV particles were purified by means of discontinuous sucrose density gradient ultracentrifugation and stored in TNE buffer as aliquots at -80°C. Before use, all rSFV particles were activated by the addition of 1:20 volume 10 mg/ml α-chymotrypsin (Sigma Chemical, St. Louis, US) and 2 mM CaCl_2_ for 30 minutes to cleave the mutated spike proteins. After which, the α-chymotrypsin was inactivated by the addition of 1:2 volume 2 mg/ml aprotinin (Sigma Chemical).

#### Titer determination of rSFV-particles

Titer determination of rSFV-particles was performed as described previously.^45^ Briefly, rSFV-particles were titrated by serial dilution on monolayers of BHK-21 cells (45000 cells) cultured in LabTek slides. After infection and incubation for 24 hours, the cells were fixed in 10% (w/v) acetone and further stained for nsP3 using a primary polyclonal rabbit-anti-nsP3 antibody (1:2000 dilution), whilst a secondary Cy3-labeled animal-anti-rabbit antibody (1:200 dilution) was used to amplify the signal. Positive cells were counted using fluorescence microscopy, and the titers were determined after considering the dilution factor.

#### Incucyte-based microscopy to quantify infectivity of rSFV-particles

LNCaP and PANC-1 cells were plated overnight (10,000 cells per well in 96 well plates) and then infected with different multiplicity of infection (MOI) of rSFV-GFP particles. Incucyte-based brightfield and fluorescence microscopy was performed over time to assess the morphology and infectivity respectively. The number of GFP-expressing cells in an image was used as a measure of rSFV-GFP infectivity.

#### Validation of cytokine expression and secretion in supernatant

LNCaP and PANC-1 cells were plated overnight (40,000 cells per well in 48 well plate) and then infected with MOI-10 of different rSFV-particles. Supernatants from cell culture were collected at 6, 24, and 48 hours post-infection and stored at -20°C for further testing. Enzyme-linked immunosorbent assay (ELISA) was performed using the supernatants to quantify the amount of secreted CXCL10 (439904, BioLegend), IFN-ƴ (430101, BioLegend) and Flt3L (DFK00, RnD Systems) using kits according to the suppliers’ protocol.

#### Monolayer based transwell assay for immune recruitment

LNCaP and PANC-1 cells were plated overnight (400,000 cells per well in 6 well plates) and then infected with MOI-10 of different rSFV-particles. Supernatants from cell cultures were collected at 24 hours post-infection and stored at -20°C for further testing. Transwells of 3 μm pore size (662630, Greiner) were used to set up the migration assay in a 24-well plate. The bottom chamber was filled with 500 μl supernatant collected from infected LNCaP or PANC-1 cells. Freshly thawed peripheral blood mononuclear cells (PBMC) from HLA-2 typed healthy donors were resuspended in complemented media containing 5% serum and added in the transwell top chamber (500,000 cells per insert). Migration was immediately quantified using Incucyte-based brightfield microscopy. The number of migrated cells was counted using the Incucyte-analysis software.

#### Monolayer-based co-culture assay for T cell activation

LNCaP and PANC-1 cells were plated overnight (100,000 cells per well in 24 well plates) and then infected with MOI-10 of different rSFV-particles. The replicon particles remaining in the supernatant were washed away 24 hours post-infection in order to focus on immune activation mediated by infected cancer cells and not directly by replicon particles. Freshly thawed PBMC from HLA-2A-typed healthy donors were co-cultured with the infected cancer cells for 24 hours. Later, cells were collected and processed for flow cytometry-based analysis of CD4 and CD8 -T cell populations for activation markers.

#### Antibodies used for flow cytometry

**Table:** 

Antibody target	Company	#Catalog
PE anti-human CD223 (LAG-3) 11C3C65	Biolegend	369306
Brilliant Violet 421™ anti-humanCD152 (CTLA-4) BNI3	Biolegend	369606
APC anti-human CD279 (PD-1) A17188B	Biolegend	621610
PE anti-human CD107a H4A3	Biolegend	328608
APC anti-human CD69 FN50	Biolegend	310910
Brilliant Violet 421™ anti-human CD38 HIT2	Biolegend	303526
APC/Cyanine7 anti-human CD8 SK11	Biolegend	344714
FITC anti-human CD4 OKT4	Biolegend	317408

#### Characterization of rSFV-infection in cancer-spheroids

LNCaP and PANC-1 cells were plated overnight (10,000 cells per well in Nunclon Sphera 96 well plates, 174925, ThermoScientific). To ensure spheroid formation, the plate was centrifuged at 1500 RPM for 10 mins at 20°C before overnight incubation. The spheroids were infected with different MOI of rSFV-GFP particles to quantify infectivity. Incucyte-based brightfield and fluorescence microscopy were performed over time to assess the morphology and infectivity of the spheroid respectively. The number of GFP-expressing cells in an image was used as a measure of rSFV-GFP infectivity. Confocal fluorescence microscopy was performed using the CD7 platform to quantify the depth at which rSFV-GFP particles can infect cells in the spheroid.

#### Spheroid-based co-culture assay for immune penetration and association

LNCaP and PANC-1 spheroids were infected with different rSFV-particles. 24 hours post-infection, freshly thawed PBMC from HLA-2A-typed healthy donors were first labeled with Celltrace FarRed (C34572, Invitrogen) and then co-cultured (100,000 cells per well) with the infected spheroids. PBMC association and penetration in the spheroid were quantified over time using Incucyte-based fluorescence microscopy. FarRed positive cells present in or on the spheroids were counted per image for quantifying the degree of PBMC penetration and association.

#### Spheroid-based co-culture assay for T cell activation

LNCaP and PANC-1 spheroids were infected with different rSFV-particles. 24 hours post-infection, freshly thawed PBMCs from HLA2A-typed healthy donors (100,000 cells per well) were added to the infected spheroids. After 24 hours of co-culture, PBMC from the culture suspension were collected separately. Simultaneously, the spheroids were collected and pooled (6 spheroids per replicate) to have enough events for flow cytometry. Spheroid samples were washed with PBS to remove loosely attached PBMC. Subsequently, the spheroids were dissociated using Trypsin-EDTA for 2 minutes to collect PBMC associated with spheroids. Finally, PBMC from suspension and PBMC associated with spheroids were stained for CD4 and CD8 -T cell populations along with immune activation markers for flow cytometry analysis.

#### Blocking of interferon pathway in PANC-1 cells with Ruxolitinib

Overnight cultured PANC-1 cells were treated with 400nM or 2μM Ruxolitinib (Stem Cell technologies) for 2 hours before infection with rSFV-particles. Next, rSFV-particles were resuspended in fresh media along with the required concentration of Ruxolitinib and added to the PANC-1 cells for infection. 24 hours post-infection the media was removed and cells were washed with PBS before adding PBMCs with fresh media for a co-culture assay.

#### RT-qPCR analysis of antiviral gene expression in LNCaP and PANC-1 cells

Overnight cultured LNCaP or PANC-1 cells were infected with MOI-10 of rSFV-GFP replicon particles. 24 hours post infection, cells were harvested and processed for RNA isolation by TRIzol reagent (ThermoFischer) according to the recommended protocol. cDNA was synthesized using 1 μg of isolated RNA (Takara-Bio) and Real-Time PCR was performed using SyBR Green chemistry (Takara-Bio) for quantification. For each sample the real time PCR was performed in technical duplicates. Gene expression levels were normalized to the expression of The relative gene expression (fold change) was assessed by the comparative Ct (2^-ΔΔCt^) method, according to Livak and Schmittgen,^46^ where expression of glyceraldehyde 3-phosphate dehydrogenase was used as endogenous control and the uninfected cells were used as reference sample.

#### Primers used for rt-qPCR for antiviral genes

**Table:** 

Target gene	Primer sequence
nfkb1	Forward: GCAGCACTACTTCTTGACCACC Reverse: TCTGCTCCTGAGCATTGACGTC
stat1	Forward: ATGGCAGTCTGGCGGCTGAATT Reverse: CCAAAACCAGGCTGGCACAATTG
ddx58 (RIG-I)	Forward: CACCTCAGTTGCTGATGAAGGC Reverse: GTCAGAAGGAAGCACTTGCTACC
tlr3	Forward: GCGCTAAAAAGTGAAGAACTGGAT Reverse: GCTGGACATTGTTCAGAAAGAGG
myd88	Forward: GAGGCTGAGAAGCCTTTACAGG Reverse: GCAGATGAAGGCATCGAAACGC
cxcl10	Forward: GGTGAGAAGAGATGTCTGAATCC Reverse: GTCCATCCTTGGAAGCACTGCA
ifn-beta1	Forward: GGTTACCTCCGAAACTGAAGA Reverse: CCTTTCATATGCAGTACATTAGCC
gapdh	Forward: GTCTCCTCTGACTTCAACAGCG Reverse: ACCACCCTGTTGCTGTAGCCAA

### Quantification and statistical analysis

Experimental data represents the mean ± SEM of the number of replicates. Significance was determined by one-way or two-way analysis of variance (ANOVA) followed by a Boneferroni post hoc test using Graphpad Prism 9 (GraphPad. San Diego, CA, USA). A *p* value of 0.05 was considered a statistically significant difference between compared groups (∗ = p < .05, ∗∗ = p < .01 and ∗∗∗ = p < .001). Graphs were made using Rstudio and Graphpad Prism 9.
