# Supplementary material for: Oncolytic alphavirus replicons mediated recruitment and activation of T cells
Source: iScience. 2024 Feb 16;27(3):109253. doi: 10.1016/j.isci.2024.109253 (PMC10904282; doi:10.1016/j.isci.2024.109253)
Supplement: Document S1. Figures S1–S10 [file mmc1.pdf]

iScience, Volume *27*

Supplemental information

Oncolytic alphavirus replicons mediated recruitment and activation of T cells

Darshak K. Bhatt, Saskia L. Meuleman, Baukje Nynke Hoogeboom, and Toos Daemen

Supplementary data

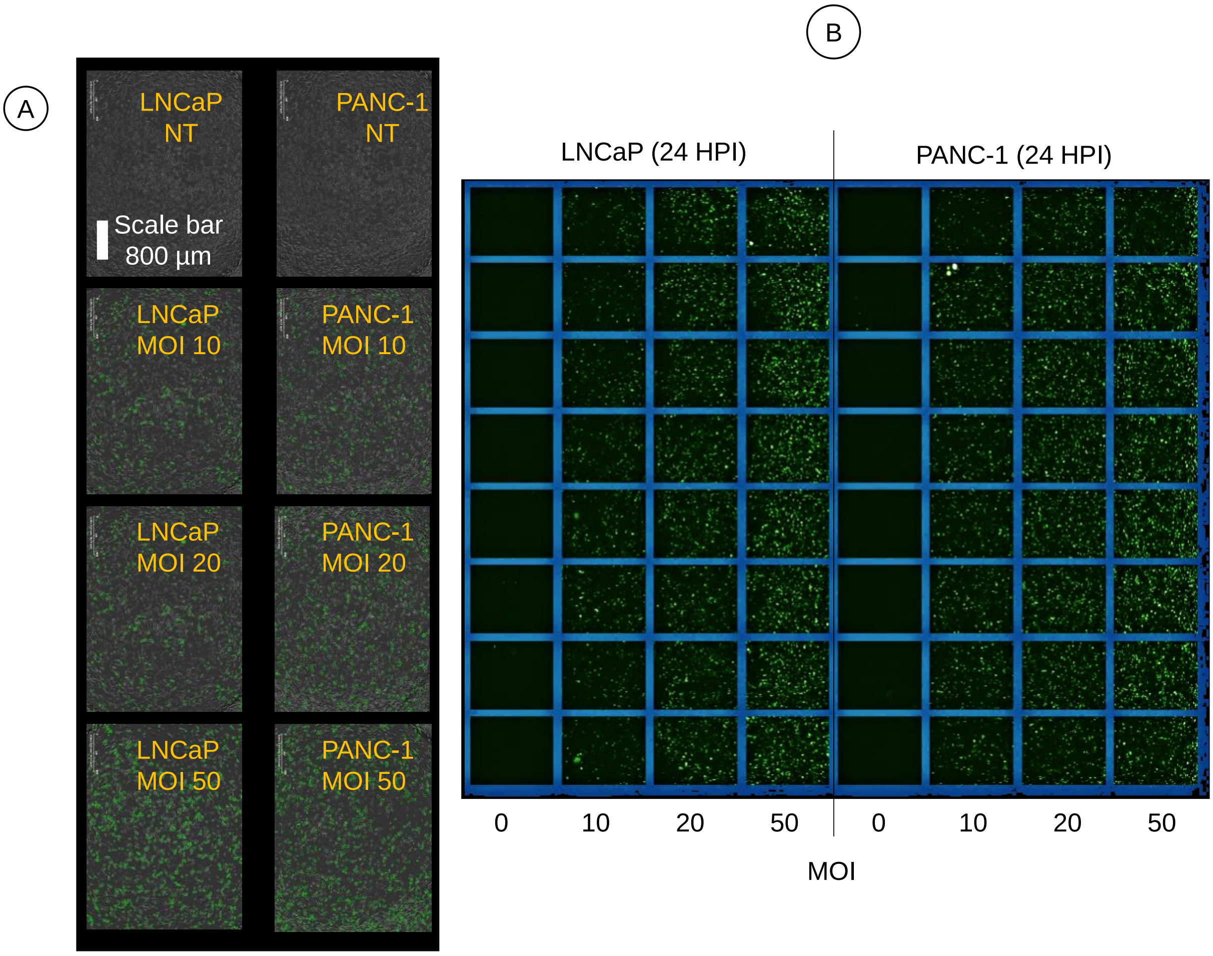

Supplementary Figure 1: Microscopic visualization of transgene expression by rSFV-particles. The microscopy images correspond to Figure 1. (A) LNCaP or PANC-1 cells in monolayer expressing GFP upon infection with different MOI of rSFV-GFP particles. (B) Visualizing 8 replicates (in rows) for each cell line and condition.

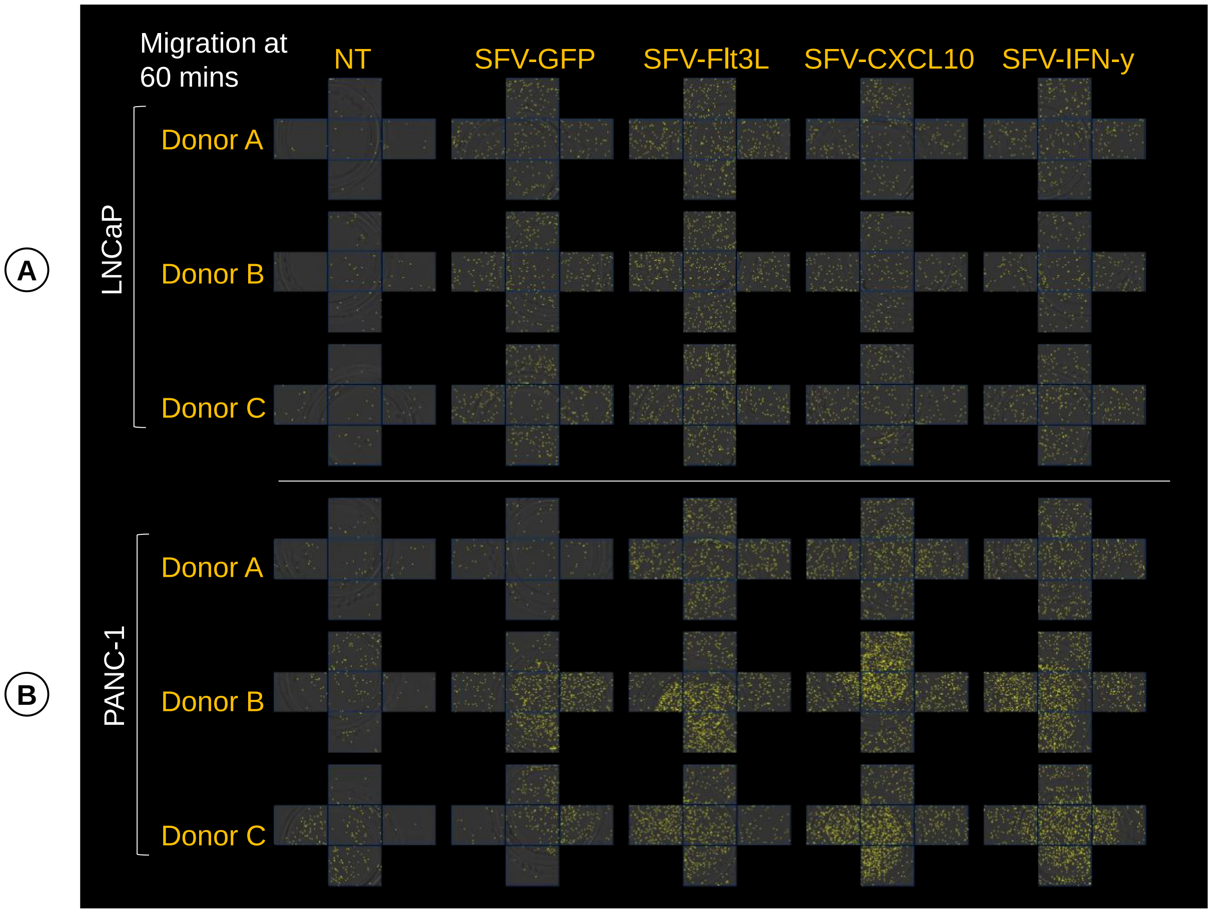

Supplementary Figure 2: Microscopy-based quantification of PBMC migration in a transwell assay. The microscopy images correspond to Figure 2 and represent migrated PBMC (marked in yellow dots) towards supernatants from infected or non-infected (A) LNCaP cells and (B) PANC-1 cells as quantified from the brightfield images using the Incucyte-analysis software. Three healthy PBMC donors were used for the experiment to assess donor-dependent heterogeneity in migration response.

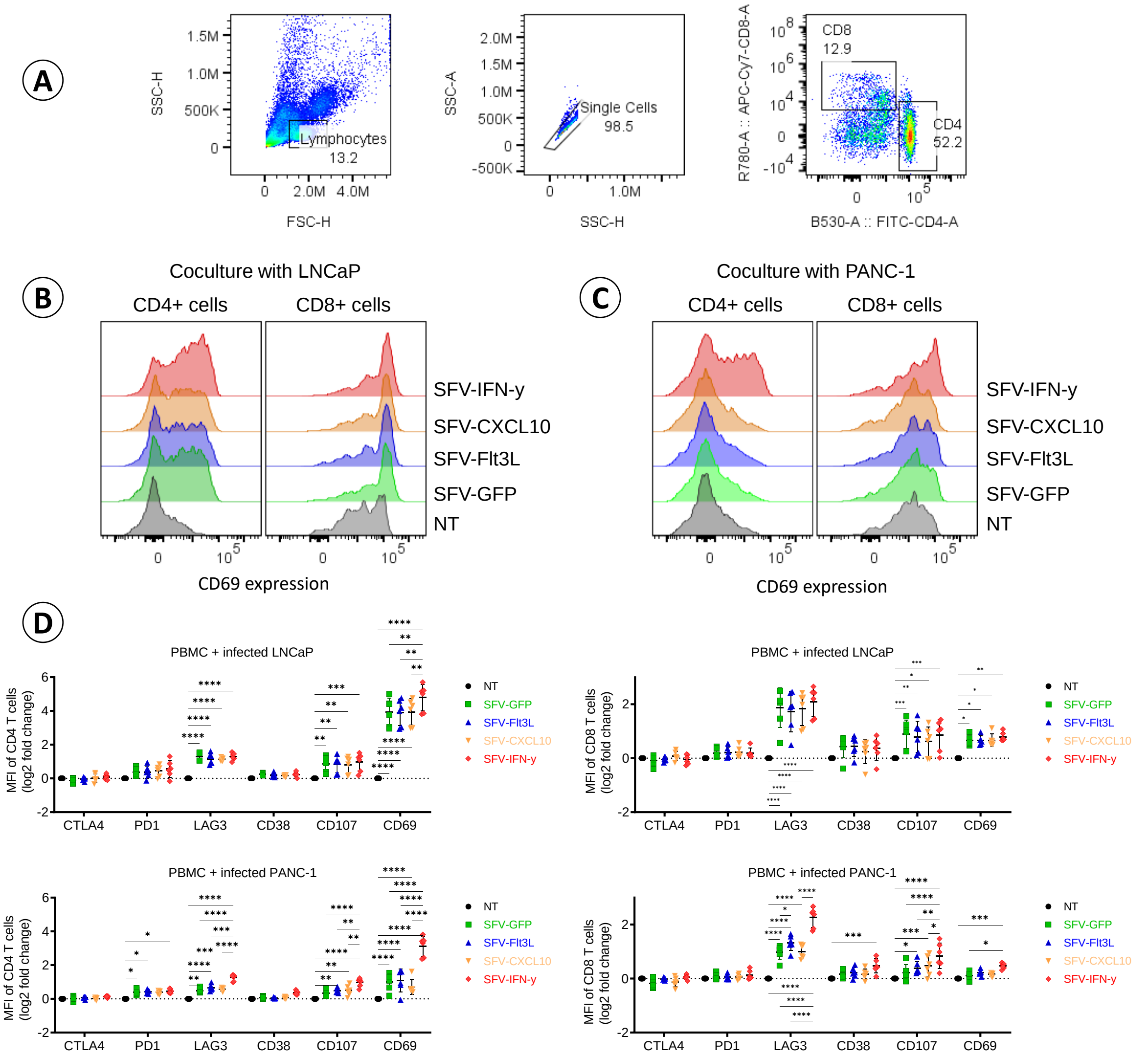

Supplementary Figure 3: Immune activation in response to rSFV-infected cancer cells. (A) The gating strategy to analyze expression of activation and exhaustion markers in CD4+ and CD8+ T cells after 24 hours of co-culture. CD69 expression by CD4 and CD8 T cells from PBMC cocultured with infected or non-infected LNCaP (B) or PANC-1 (C) cells. (D) The plots correspond to Figure 3 and depict protein level expression of activation and exhaustion markers in CD4+ and CD8+ T cells. The median fluorescence intensity (MFI) of each marker is normalized to the NT condition, i.e. PBMC co-culture with un-infected cancer cells. The MFI is represented in log2 scale. In (D) each dot represents a replicate, with total 6 replicates derived from a duplicate condition of 3 healthy donors.

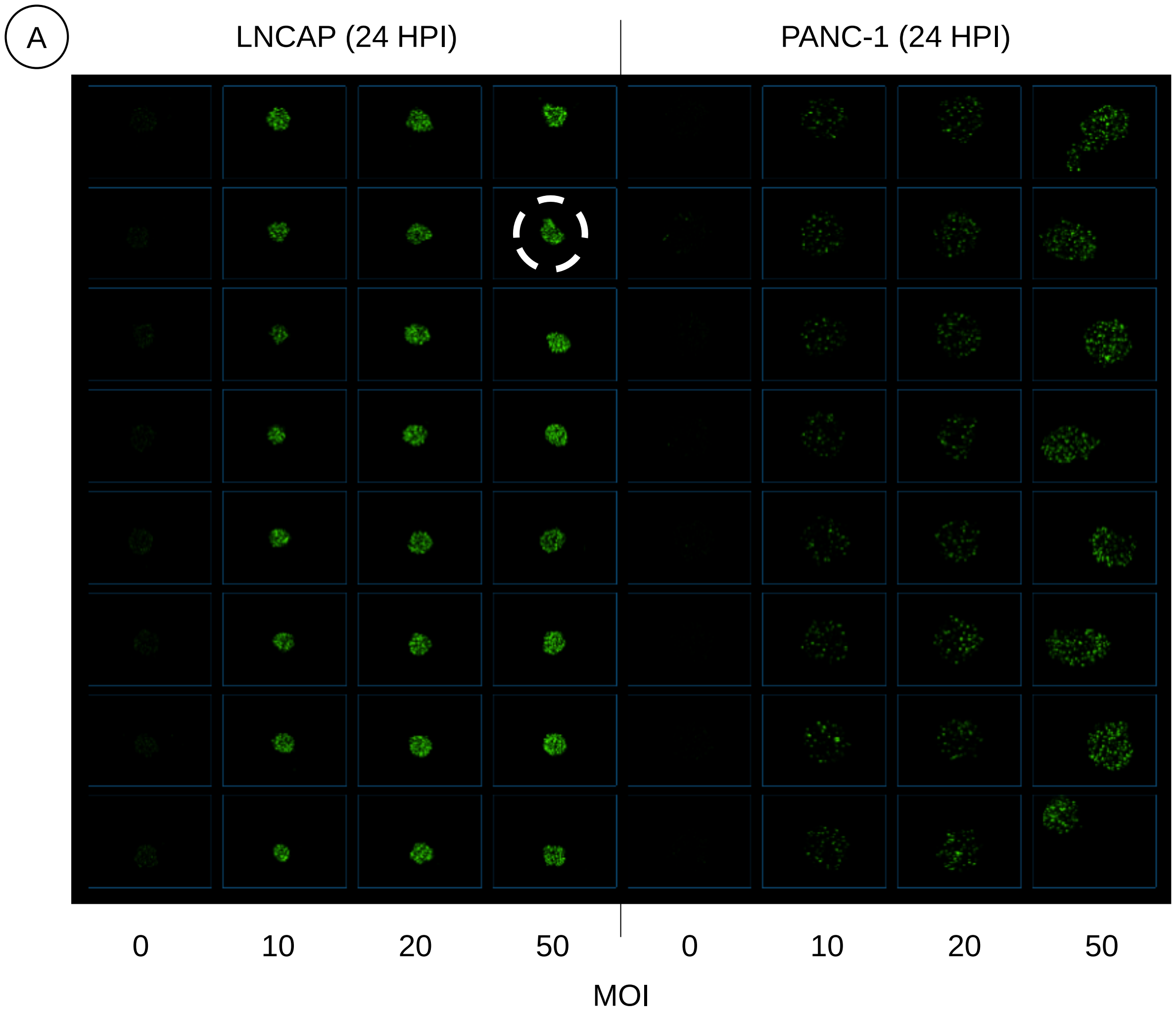

Supplementary Figure 4: Transgene expression by rSFV-particles in cancer-spheroids. The microscopy images correspond to Figure 4. (A) Fluorescence channel images to visualize GFP expression by LNCaP (left) or PANC-1 (right) spheroids upon infection with different MOI of rSFV-GFP particles after 24 hours of infection. The 8 replicates are arranged in rows. The LNCaP spheroid in the second row infected with MOI50 of rSFV-GFP particles was used for confocal microscopy as described in Figure 4D.

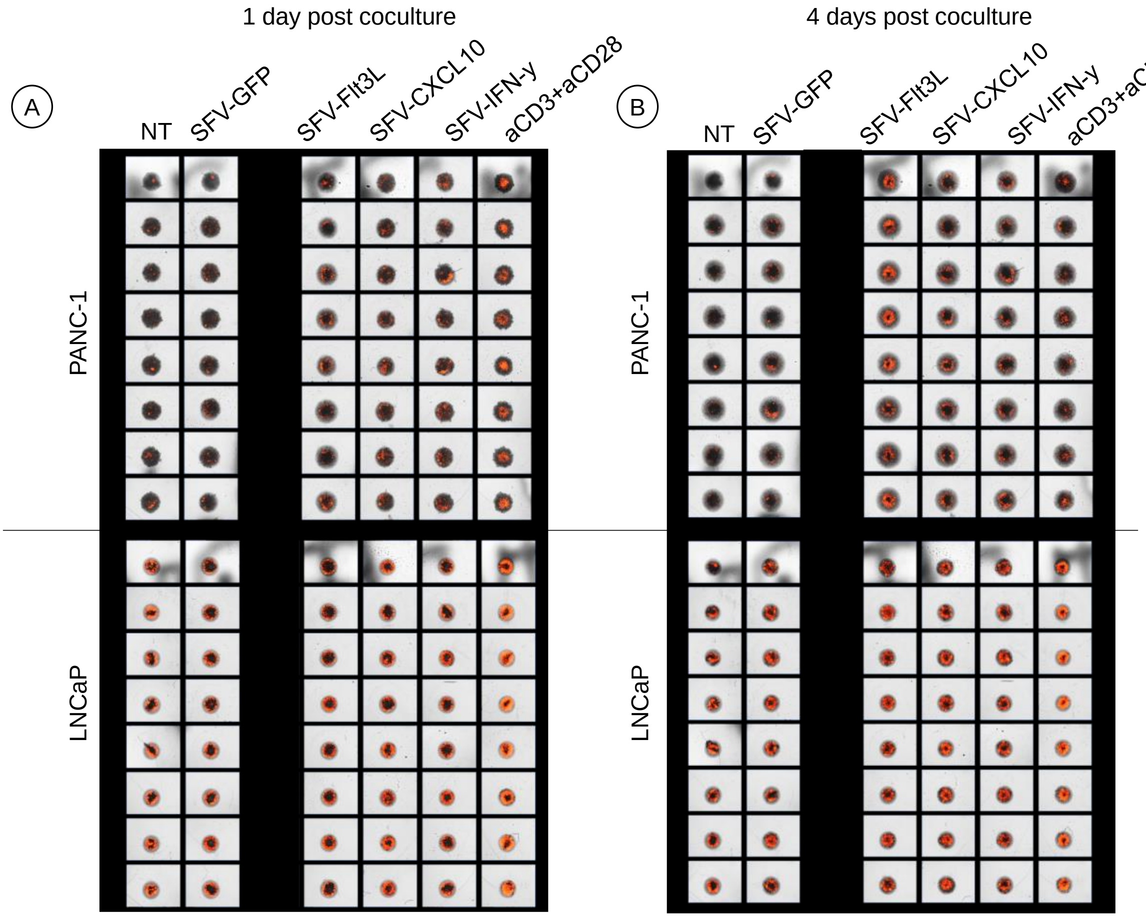

Supplementary Figure 5: Immune cell infiltration in cancer spheroids by rSFV particle-mediated infection and cytokine expression. The microscopy images correspond to data from Figure 5 and depict PBMC (in red) associated with PANC-1 (top row) or LNCaP (bottom row) spheroids at (A) 1 day and (B) 4 days post coculture with infected or non-infected spheroids. The 8 replicates for each condition are represented in respective rows.

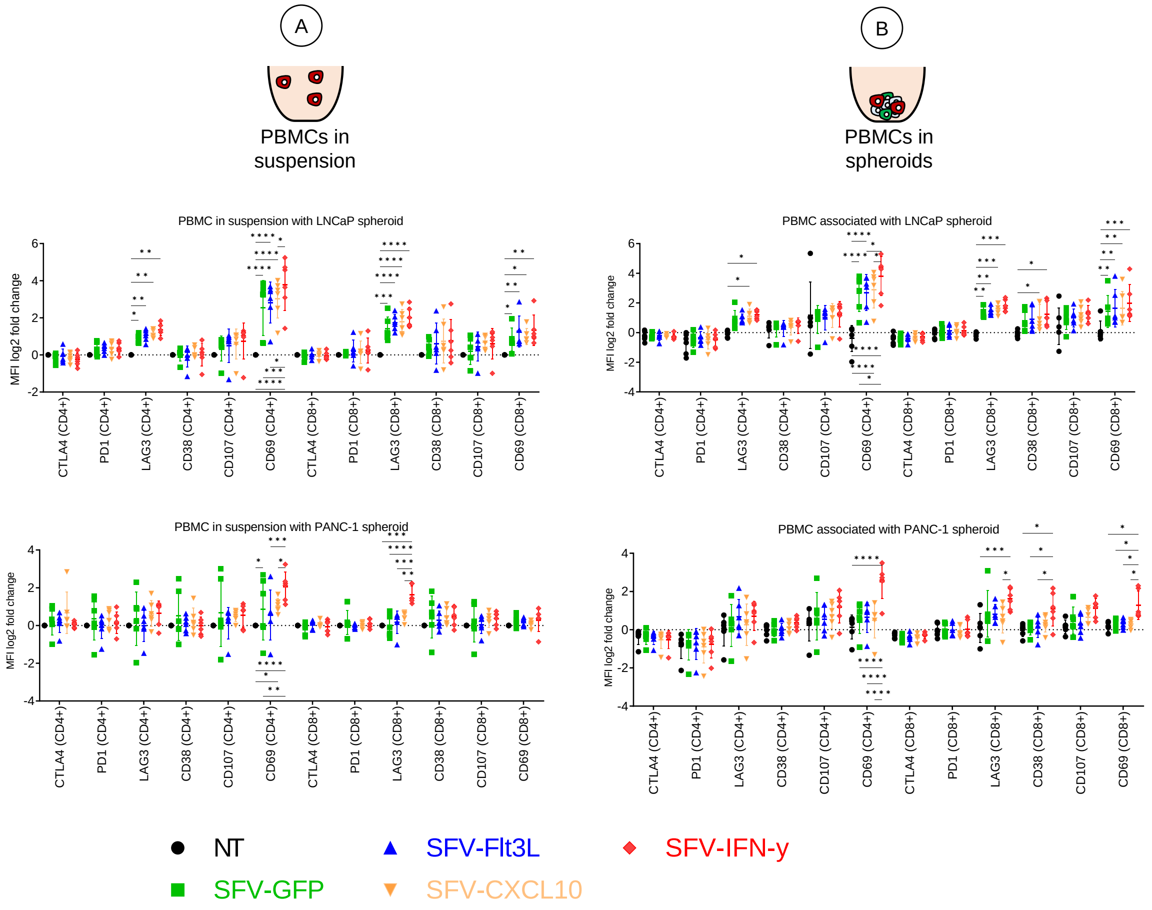

Supplementary Figure 6: Immune activation by rSFV-infected cancer-spheroids. The plots correspond to the data from Figure 6 and depict protein level expression of activation and exhaustion markers in CD4+ and CD8+ T cells present in suspension (A) or associated with spheroids (B). The median fluorescence intensity (MFI) of each marker is normalized to the NT condition from T cells in suspension. The MFI is represented in log2 scale. In all the plots each dot represents a replicate, with total 6 replicates derived from duplicate condition of 3 healthy donors.

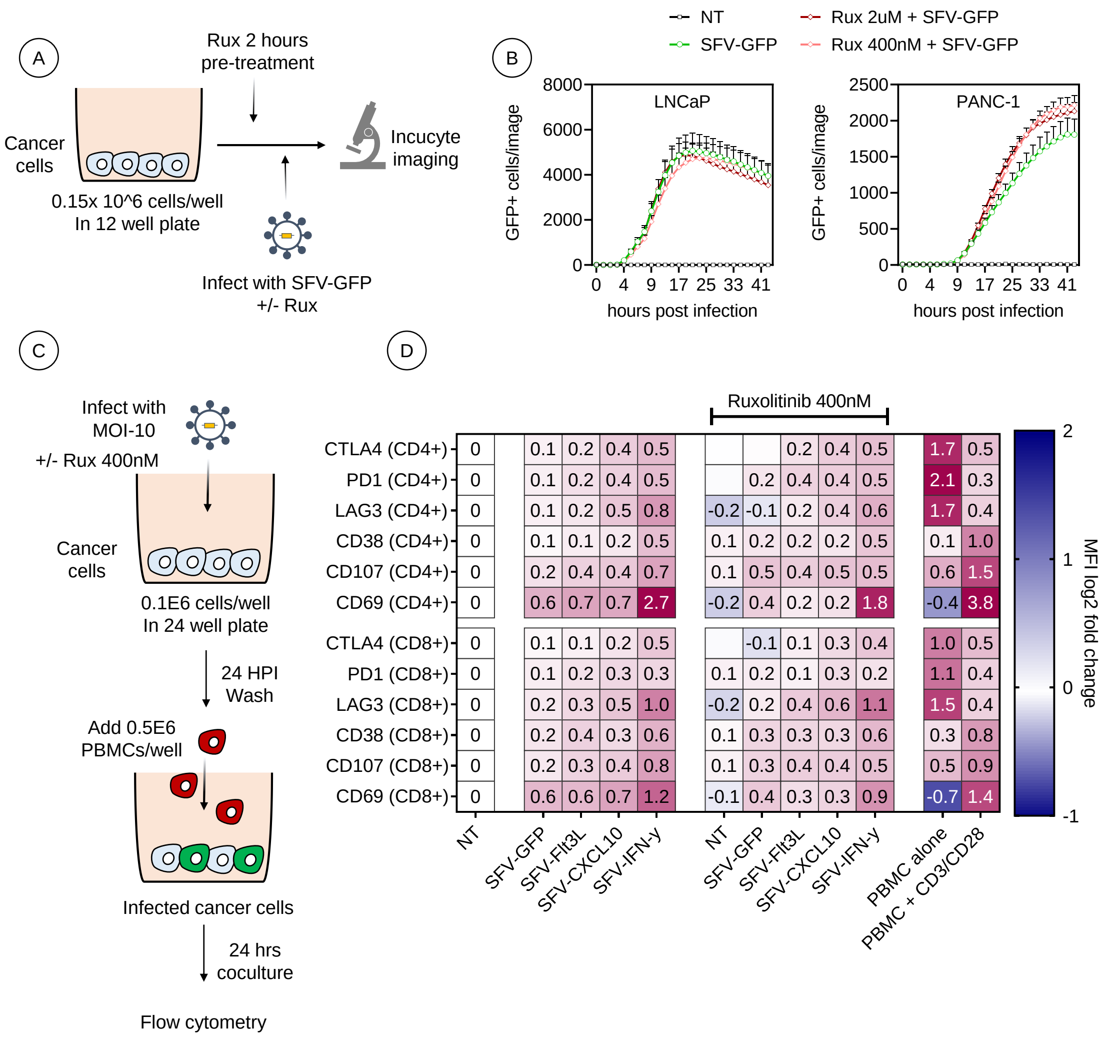

Supplementary Figure 7: Immune activation by PANC-1 cells infected by rSFV-particles in presence of interferon signaling inhibitor. (A) The setup of a monolayer-based infection assay to assess the magnitude of sensitization of PANC-1 cells by Ruxolitinib. (B) Temporal kinetics of GFP expression by LNCaP and PANC-1 cell lines infected with SFV-GFP replicon particles in the presence of Ruxolitinib at different concentrations. (C) The setup of a monolayer-based co-culture assay of PANC-1 cells infected in presence of Ruxolitinib and PBMC to assess immunogenic potential of rSFV-particles. (D) Protein level expression of exhaustion and activation markers in CD4+ or CD8+ T cells upon co-culture with infected or non-infected PANC-1 cells. Median fluorescence intensity (MFI) for each marker quantified through flow cytometry is normalized to the non-infected (NT) condition. The values in (D) are the mean expression values of different donors represented on a log2 scale. Legend: NT = non-infected cancer cells, SFV-GFP = cancer cells infected with rSFV encoding GFP, SFV-Flt3L = cancer

cells infected with rSFV encoding Flt3L, SFV-CXCL10 = cancer cells infected with rSFV encoding CXCL10, SFV-IFN-γ = cancer cells infected with rSFV encoding IFN-γ, PBMC alone = PBMC in absence of PANC-1 cells, PBMC + CD3/CD28 = PBMCs treated with anti-CD3 and anti-CD28 antibody. In (D) the plots represent data from duplicate conditions of 3 independent healthy donors. This figure corresponds to figure 1, figure 3 and figure 6.

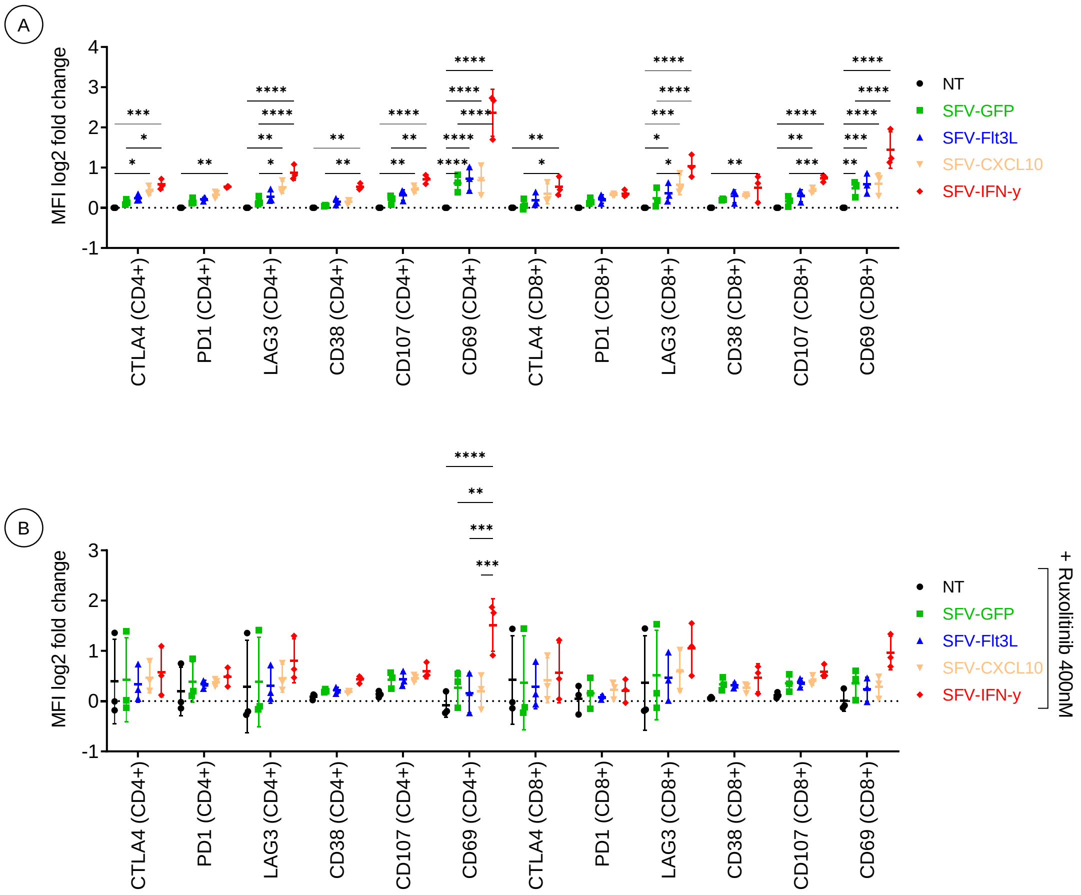

Supplementary Figure 8: Immune activation by rSFV-infected PANC-1 cells in the presence of Ruxolitinib. The plots correspond to the data from supplementary Figure 7 and depict protein level expression of activation and exhaustion markers in CD4+ and CD8+ T cells from PBMCs in coculture with PANC-1 cells infected in the absence (A) or presence (B) of Ruxolitinib. The median fluorescence intensity (MFI) of each marker is normalized to the NT condition from PBMCs in coculture with PANC-1 cells. The MFI is represented in log2 scale. In both plots each dot represents a replicate, with a total of 6 replicates derived from duplicate conditions of 3 healthy donors.

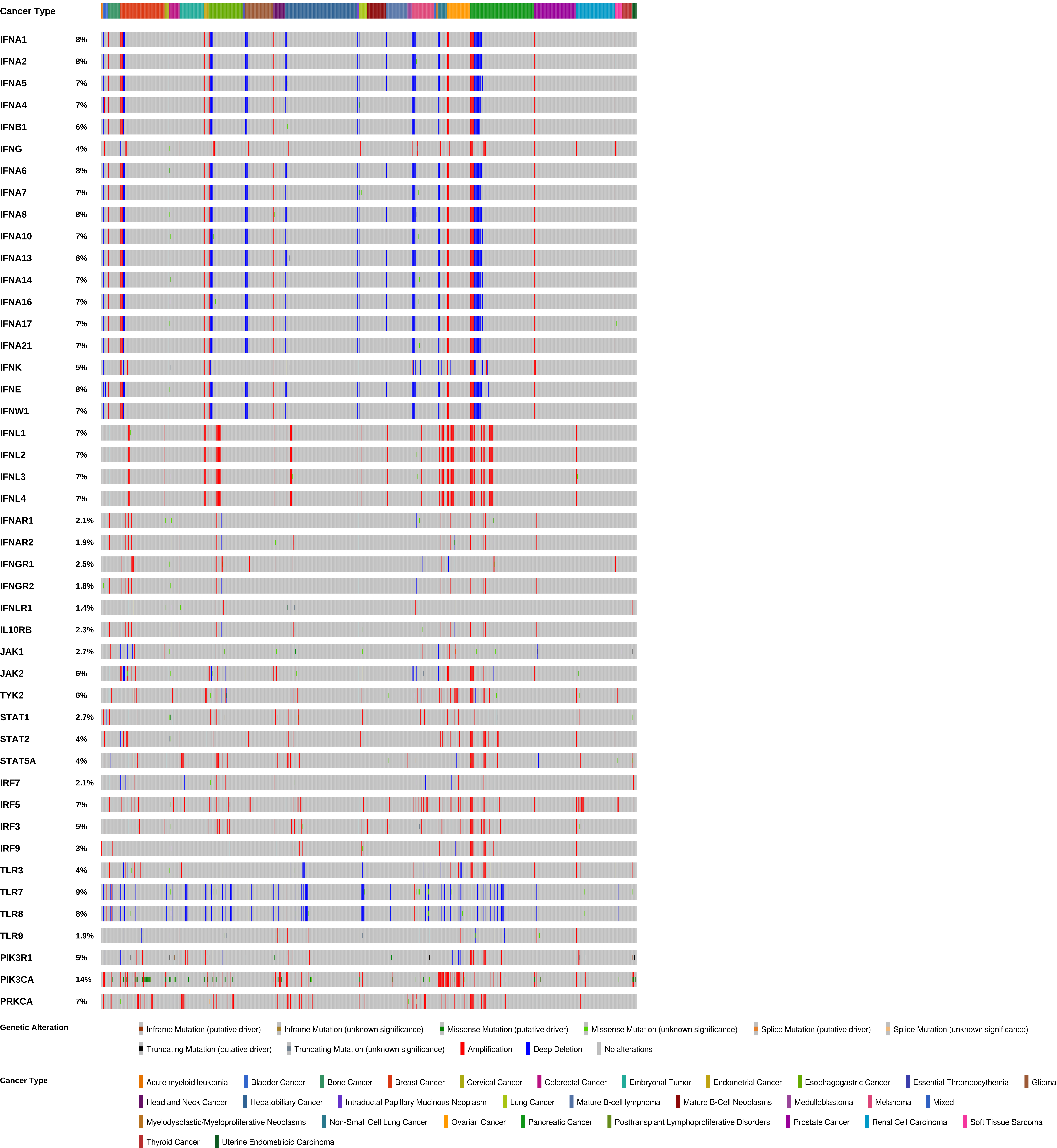
Cancer Type
IFNA1 8%
IFNA2 8%
IFNA5 7%
IFNA4 7%
IFNB1 6%
IFNG 4%
IFNA6 8%
IFNA7 7%
IFNA8 8%
IFNA10 7%
IFNA13 8%
IFNA14 7%
IFNA16 7%
IFNA17 7%
IFNA21 7%
IFNK 5%
IFNE 8%
IFNW1 7%
IFNL1 7%
IFNL2 7%
IFNL3 7%
IFNL4 7%
IFNAR1 2.1%
IFNAR2 1.9%
IFNGR1 2.5%
IFNGR2 1.8%
IFNLR1 1.4%
IL10RB 2.3%
JAK1 2.7%
JAK2 6%
TYK2 6%
STAT1 2.7%
STAT2 4%
STAT5A 4%
IRF7 2.1%
IRF5 7%
IRF3 5%
IRF9 3%
TLR3 4%
TLR7 9%
TLR8 8%
TLR9 1.9%
PIK3R1 5%
PIK3CA 14%
PRKCA 7%
Genetic Alteration
Inframe Mutation (putative driver)
Inframe Mutation (unknown significance)
Missense Mutation (putative driver)
Missense Mutation (unknown significance)
Splice Mutation (putative driver)
Splice Mutation (unknown significance)
Truncating Mutation (putative driver)
Truncating Mutation (unknown significance)
Amplification
Deep Deletion
No alterations
Cancer Type
Acute myeloid leukemia
Bladder Cancer
Bone Cancer
Breast Cancer
Cervical Cancer
Colorectal Cancer
Embryonal Tumor
Endometrial Cancer
Esophagogastric Cancer
Essential Thrombocythemia
Glioma
Head and Neck Cancer
Hepatobiliary Cancer
Intraductal Papillary Mucinous Neoplasm
Lung Cancer
Mature B-cell lymphoma
Mature B-Cell Neoplasms
Medulloblastoma
Melanoma
Mixed
Myelodysplastic/Myeloproliferative Neoplasms
Non-Small Cell Lung Cancer
Ovarian Cancer
Pancreatic Cancer
Posttransplant Lymphoproliferative Disorders
Prostate Cancer
Renal Cell Carcinoma
Soft Tissue Sarcoma
Thyroid Cancer
Uterine Endometrioid Carcinoma

Supplementary Figure 9: Pan-cancer analysis of whole genomes (ICGC/TCGA, Nature 2020) to look for mutations in genes regulating interferon-pathway. Samples from 2565 patients with different cancer backgrounds. The query used to access the data can be retrieved on this link: https://bit.ly/3KIztW3. This figure corresponds to figure 1, figure 3 and figure 6.

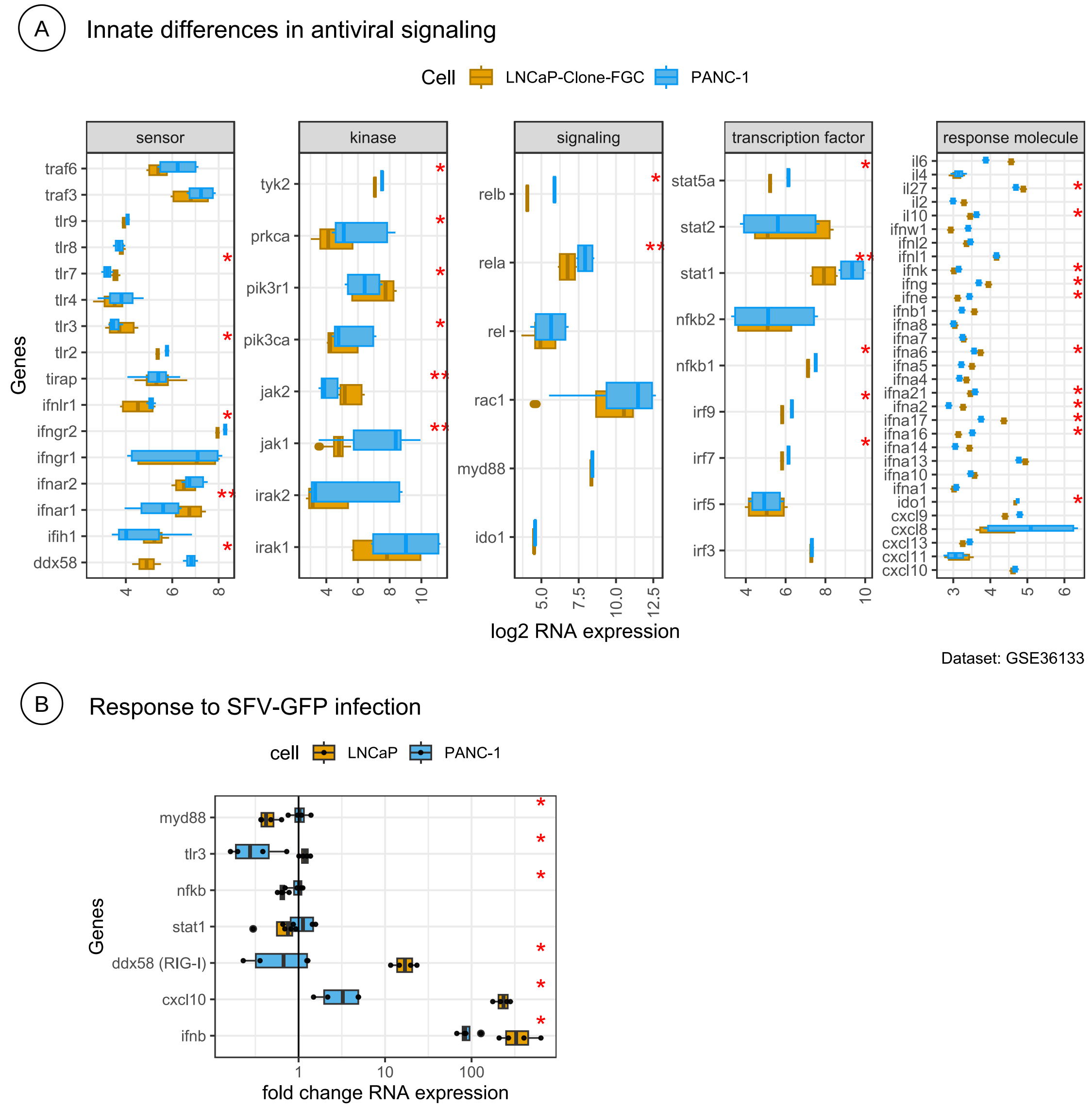

Supplementary figure 10: Innate differences in antiviral signaling between LNCaP and PANC1 cells. Variation in expression of genes related to antiviral signaling pathway between (A) untreated LNCaP and PANC-1 cells, and (B) SFV-GFP infected LNCaP and PANC-1 cells. The GSE36133 dataset was used for figure (A). In (B) LNCaP or PANC-1 cells were infected with SFV-GFP virus (MOI = 10). 24 hours post infection, the cells were processed for RNA isolation using TRIzol according to manufacturer's instructions (Thermo Fischer Scientific). cDNA was synthesized using 1 µg of isolated RNA (Takara-Bio) and Real-Time PCR was performed using SyBR Green chemistry (Takara-Bio) for quantification. In the plot, each dot represents a replicate, with a total of 4 replicates derived from two biological replicates and two technical replicates. This figure corresponds to figure 1, figure 3 and figure 6.
